# QTL detection for coccidiosis (*Eimeria tenella*) resistance in a Fayoumi × Leghorn F_2_ cross, using a medium-density SNP panel

**DOI:** 10.1186/1297-9686-46-14

**Published:** 2014-02-19

**Authors:** Nicola Bacciu, Bertrand Bed’Hom, Olivier Filangi, Hélène Romé, David Gourichon, Jean-Michel Répérant, Pascale Le Roy, Marie-Hélène Pinard-van der Laan, Olivier Demeure

**Affiliations:** 1 INRA, UMR 1348 PEGASE, Domaine de la Prise, F-35590 Saint-Gilles, France; 2 Agrocampus Ouest, UMR 1348 PEGASE, Domaine de la Prise, F-35590 Saint-Gilles, France; 3 INRA, UMR 1313 Génétique Animale et Biologie Intégrative, F-78352 Jouy-en-Josas, France; 4 Pôle d’expérimentation avicole de Tours, INRA, F-37380 Nouzilly, France; 5 UR VIPAC, ANSES, F-22440 Ploufragan, France

## Abstract

**Background:**

Coccidiosis is a major parasitic disease that causes huge economic losses to the poultry industry. Its pathogenicity leads to depression of body weight gain, lesions and, in the most serious cases, death in affected animals. Genetic variability for resistance to coccidiosis in the chicken has been demonstrated and if this natural resistance could be exploited, it would reduce the costs of the disease. Previously, a design to characterize the genetic regulation of *Eimeria tenella* resistance was set up in a Fayoumi × Leghorn F_2_ cross. The 860 F_2_ animals of this design were phenotyped for weight gain, plasma coloration, hematocrit level, intestinal lesion score and body temperature. In the work reported here, the 860 animals were genotyped for a panel of 1393 (157 microsatellites and 1236 single nucleotide polymorphism (SNP) markers that cover the sequenced genome (i.e. the 28 first autosomes and the Z chromosome). In addition, with the aim of finding an index capable of explaining a large amount of the variance associated with resistance to coccidiosis, a composite factor was derived by combining the variables of all these traits in a single variable. QTL detection was performed by linkage analysis using GridQTL and QTLMap. Single and multi-QTL models were applied.

**Results:**

Thirty-one QTL were identified i.e. 27 with the single-QTL model and four with the multi-QTL model and the average confidence interval was 5.9 cM. Only a few QTL were common with the previous study that used the same design but focused on the 260 more extreme animals that were genotyped with the 157 microsatellites only. Major differences were also found between results obtained with QTLMap and GridQTL.

**Conclusions:**

The medium-density SNP panel made it possible to genotype new regions of the chicken genome (including micro-chromosomes) that were involved in the genetic control of the traits investigated. This study also highlights the strong variations in QTL detection between different models and marker densities.

## Background

Coccidia are animal parasites that are present mainly in the intestines and cause coccidiosis. Coccidia are sub-classified into several genera, including *Eimeria*, *Isospora*, *Cryptosporidium*, *Toxoplasma* and *Sarcocystis*. Here, the term ‘coccidiosis’ refers exclusively to the genus *Eimeria* that is the major cause of parasitic avian coccidiosis with huge economic losses to the poultry industry. Estimates put the annual worldwide cost of coccidiosis to poultry production at up to 2 billion dollars [1]. *Eimeria tenella*, which develops in the cecum, is one of the most frequent coccidian species involved in chicken coccidiosis. It causes depression of body weight gain, lesions and, in the most serious cases, death of infected animals. Progress has been made in the development of recombinant vaccines against coccidiosis but none is yet available for use in the poultry industry. Moreover, the use of anticoccidial drugs raises several issues from both an environmental and food-chain perspective and a drug resistance issue. Genetic variability for resistance to coccidiosis in the chicken has been demonstrated [2,3] and if this natural resistance could be exploited, it would reduce the costs of the disease. Using a low-density genetic map (157 microsatellites) and a single-QTL model, Pinard-van der Laan et al. [4] characterized the genetic regulation of *Eimeria tenella* resistance in a Fayoumi × Leghorn F_2_ cross (Fayoumi and Leghorn breeds are respectively resistant and susceptible to coccidiosis [5]) and identified 21 QTL (quantitative trait locus). Today, the possibility to generate high-density single nucleotide polymorphism (SNP) genotyping data and the use of more complex models that can test multiple QTL in the same linkage group have dramatically increased the power and accuracy of such analyses. In this work, we used a custom SNP array and novel quantitative models to re-analyze the Fayoumi × Leghorn F_2_ design. In addition, as one objective of this study was to find an index capable of explaining a large amount of the variance associated with resistance to coccidiosis, a composite variable was produced using the five studied traits and used for QTL detection.

## Methods

### Animals and measurements

The experimental design is detailed in [4]. Briefly, a F_1_ cross was produced using three roosters and seven hens from the Fayoumi (resistant) line and three roosters and six hens from the Leghorn (susceptible) line. Among the F_1_ animals, six roosters and 30 hens (six groups of sisters, one group per rooster) were randomly mated to produce 860 F_2_ of both sexes [4]. F_2_ animals were challenged for coccidiosis resistance at the ANSES Parasitology unit at Ploufragan. Chickens were weighed at 26 days of age, separated according to sex and dam family into similar-weight groups. Inoculation was performed at 28 days of age with a dose of 50 000 *Eimeria tenella* oocysts [4]. Challenged birds were slaughtered at 36 days of age, i.e. 8 days post-inoculation. Body weight gain (WG) was measured as WG = 100 × (body weight (8 days post-inoculation) – body weight (2 days pre-inoculation))/body weight (2 days pre-inoculation). Blood was sampled at 4 days post-inoculation to measure plasma coloration (PC, as a measure of the level of blood carotenoid) as PC = log_10_(optical density at 480 nm) and hematocrit level (HEMA). Rectal body temperature (T°) was measured 4 days post-inoculation. At slaughter, cecal lesion scores (LES) were assessed by the same skilled pathologist on a scale of 0 (no lesion) to 4 (most severe lesions) [6]. Briefly, the duodenum and jejunum were extracted and anonymized before the pathologist opened them. The score was assessed based on the mucosa structure conservation and blood clot content. All procedures were conducted under Licence No. 37–123 from the Veterinary Services, Indre-et-Loire, France and in accordance with guidelines for Care and Use of Animals in Agricultural Research and Teaching (French Agricultural Agency and Scientific Research Agency).

### Genotype data and construction of the genetic map

F_1_ sires were first genotyped for a set of 9216 SNPs. This preliminary step enabled us to select fully informative markers to genotype the F2 individuals i.e. 1536 SNPs that were distributed over all the sequenced genome were selected based on informativity and location using MarkerSet [7]. The 157 microsatellites already genotyped in the previous approach were included in the dataset. MendelSoft [8] was used to correct data for possible Mendelian genetics problems. Of the 1693 markers (1536 SNPs and 157 microsatellites), 1393 were kept for further analysis [see Additional file 1: Table S1] and 296 markers were eliminated due to technical problems (call rate lower than 85% and/or Mendelian errors higher than 5%).

The chicken linkage consensus map constructed by Groenen et al. [9] was used to determine the genetic location of the markers. Locations of markers unavailable in the consensus map were extrapolated based on flanking markers. Local ratio between genetic (cM) and physical distances (Mb) between two flanking markers a and b [(cM_a_ – cM_b_)/(Mb_a_ – Mb_b_)] was used to extrapolate the genetic location of a marker m by taking into consideration its physical distance from the previous marker cM_m_ = cM_a_ + [(Mb_m_ – Mb_a_) × ratio].

### Factor analysis

A fundamental model capable of explaining observed associations among variables might be suitable, potentially a latent variable model [10]. A factor analysis for all the original traits (resistance criteria) was performed, and one first-order factor termed composite variable F1 was extracted using *Proc Factor* by *SAS*[11].

### QTL mapping methods

Single-QTL detection analyses were run using GridQTL [12] and QTLMap software [13]. To be able to make comparisons with the previous study [4], the F_2_ model was used in GridQTL. For QTLMap, an interval mapping method was applied and no assumptions about either the fixation of the alleles in the founder lines or the number of segregating alleles at the QTL were made. A mixture of half and full-sib families was considered as pedigree structure, and only the sire meiosis was studied. For each trait, an LRT value was calculated at each cM to compare the fit of two models (i.e. the model with a QTL at the location considered *vs* the model without fitting any QTL effect). Chromosome-wide significance thresholds were evaluated through empirical calculations obtained by simulations under the null hypothesis. A total of 1000 simulations and 1000 permutations were performed for each trait × chromosome combination. Confidence intervals on QTL positions were estimated by the drop-off method [14]. Similarly to the reduction of one LOD when using LOD scores, the maximum LRT value was reduced by 3.84 (a *χ*^2^ distribution with one degree of freedom for *P* < 0.05) to determine a threshold. Region boundaries were then defined by the LRT locations crossing this threshold upstream and downstream of the peak LRT.

Multi-QTL analyses were also performed using QTLMap software, with the hypothesis that two linked QTL influenced the same trait [15]. These analyses applied two approaches. When a QTL is identified in a linkage group, the H1 hypothesis (there is one QTL in the linkage group) was compared to the H2 hypothesis (there are two QTL in the linkage group), which is particularly useful to test whether another QTL might be segregating elsewhere in the linkage group. When no QTL was detected for a linkage group × trait combination, the H0 (no QTL in the linkage group) was compared to the H2 hypothesis to test for potential segregation of two antagonistic QTL. In both cases, the two QTL locations under H2 are estimated considering all possible combinations based on a two-dimensional grid. For all these analyses, significance thresholds were determined by simulating performances under the null hypothesis of the test to obtain an empirical distribution of the LRT in accordance with the pedigree and marker information. For the two *versus* one QTL test, performances were simulated under the one QTL hypothesis. The most likely location and effect estimated in the no *versus* one QTL test were used to define QTL effect on performances.

QTL effects were calculated as the average of all absolute values for heterozygous sire effects, and expressed in trait units.

### Candidate genes

For each QTL region, genomic coordinates of confidence interval limits were determined on the basis of the chicken linkage consensus map constructed by Groenen et al. [9], as described previously. The list of genes present in each QTL region was extracted using AnnotQTL [16] on the WUGSC 2.1 (galGal3) assembly. The functions and interactions of these genes were analyzed using Ingenuity Pathway Analysis (IPA, http://www.ingenuity.com).

## Results and discussion

The 860 F_2_ animals were genotyped for 1393 genetic markers and QTL mapping was performed for the following traits: body weight gain (WG), plasma coloration (PC), hematocrit level (HEMA), rectal body temperature (T°), cecal lesion score (LES). A composite variable (F1) that represents most of the variance present in these traits was derived.

### Single-QTL detection

Single-QTL analyses using QTLMap and GridQTL detected 9 and 19 chromosome-wide significant QTL (P < 0.05), respectively (Table 1). Each of the five traits studied and the composite variable F1 were associated to at least one QTL. The confidence interval sizes ranged from 1 cM (QTL for WG on *Gallus gallus* chromosome GGA22) to 18 cM (T° on GGA18) with a mean of 5.9 cM, which is much smaller than the values usually obtained when using microsatellites only (12 cM on average between markers that flank the QTL). This illustrates the gain in location accuracy obtained by a 10-fold increase in genetic marker density. Interestingly, on GGA19 and GGA24, different QTL for some of the traits studied and the composite variable F1 mapped to the same location, which suggests the presence of pleiotropic QTL. On GGA24, the three detected QTL for LES, PC and composite variable F1 were positioned at the same location i.e. 4 cM, while on GGA19, three QTL were detected at 19 cM and one at 25 cM with various confidence interval sizes i.e. 18–20 cM containing QTL for LES and PC, 20–29 cM containing a QTL for HEMA and 18–28 cM containing a QTL for composite variable F1 (Table 1). Further multivariate analyses would be valuable to dissect more finely these two chromosomes.

**Table 1:** Results of the single-QTL analysis

**Tool**	**Trait**	**GGA**	**Location (cM)**	**Confidence interval size (cM)**	**Significance level**	**Additive effect**^**a**^ **± se**	**Heterozygous sires (/6)**	**Fayoumi origins**^**b**^
QTLMap	WG	1	203	200–206	*	7.12 ± 2.13	4	4/4
QTLMap	T°	2	247	240–247	*	0.21 ± 0.03	3	1/3
QTLMap	F1	3	227	224–240	*	0.36 ± 0.11	5	4/5
QTLMap	T°	8	58	45–63	*	0.21 ± 0.02	3	1/3
QTLMap	F1	9	39	35–43	*	0.33 ± 0.13	3	0/3
QTLMap	T°	10	77	72–88	*	0.19 ± 0.07	3	2/3
QTLMap	T°	11	15	15–25	*	0.17 ± 0.01	4	1/4
QTLMap	T°	16	-	-	*	0.18 ± 0.04	3	1/3
QTLMap	LES	22	2	0–5	*	0.22 ± 0.08	4	2/4
GridQTL	WG	1	96	93–99	**	-7.72 ± 1.74	-	-
GridQTL	T°	2	259	257–261	**	0.27 ± 0.06	-	-
GridQTL	WG	3	37	34–38	*	-7.02 ± 1.90	-	-
GridQTL	HEMA	3	128	125–129	*	-1.49 ± 0.51	-	-
GridQTL	PC	4	123	122–126	*	0.05 ± 0.05	-	-
GridQTL	HEMA	6	81	79–83	**	-1.9 ± 0.48	-	-
GridQTL	T°	7	61	59–64	*	0.2 ± 0.06	-	-
GridQTL	PC	11	44	43–45	*	-0.09 ± 0.05	-	-
GridQTL	F1	11	44	43–45	*	-0.20 ± 0.10	-	-
GridQTL	LES	18	6	5–10	*	0.23 ± 0.07	-	-
GridQTL	LES	19	19	18–20	**	-0.31 ± 0.08	-	-
GridQTL	HEMA	19	25	20–29	*	1.84 ± 0.50	-	-
GridQTL	PC	19	19	18–20	*	0.18 ± 0.05	-	-
GridQTL	F1	19	19	18–28	*	0.37 ± 0.11	-	-
GridQTL	WG	22	0	0–1	*	5.22 ± 1.76	-	-
GridQTL	F1	22	8	0–11	*	0.33 ± 0.10	-	-
GridQTL	LES	24	4	2–6	*	0.21 ± 0.07	-	-
GridQTL	PC	24	4	2–5	**	-0.10 ± 0.05	-	-
GridQTL	F1	24	4	2–5	*	-0.19 ± 0.1	-	-

* = 5% chromosome-wide; ** = 1% chromosome-wide.

^a^substitution effect expressed in trait units (F1 = composite variable in arbitrary units; HEMA = hematocrit level in %; LES = cecal lesion score in arbitrary units; PC = plasma coloration in optical density units; T° = rectal body temperature in °C; WG = body weight gain in %).

^b^number of alleles associated with high trait values in the Fayoumi line.

### Multi-QTL detection

Under the H0 *vs* H2 test, four QTL for composite variable F1 and LES were identified on GGA21 i.e. one pair at 19 and 20 cM and another pair at 25 and 24 cM (Table 2). Since in each pair the QTL were co-located, it is likely that there are only two QTL that affect LES and that their effects are also captured by the F1 composite variable. The hypothesis tested under the H0 *vs* H2 model is that two closely-located QTL but in repulsion phase (i.e. the favorable allele of the first QTL is in linkage disequilibrium with the unfavorable allele of the second QTL) are not identified by single-QTL analysis since their average effect is close to zero [17,18]. This is confirmed by the fact that the two QTL for composite variable F1 and the two QTL for LES were separated by only about 5 cM (Table 2) and showed similar antagonist effects in the six sire families analyzed (Table 3). In addition, identification of these two QTL positions on GGA21 using the H0 *vs* H2 model may also be explained by the fact that estimating two QTL effects together provides more power and more precision for the localization of each QTL [19]. An alternative H1 *vs* H2 hypothesis was tested for all chromosome × trait combinations for which a single QTL was previously detected. However, no additional QTL was identified using this strategy.

**Table 2:** Results of the multi-QTL analysis

**Trait**	**GGA**	**H0 vs H2**	**Location (cM)**		**Additive effect**^**a**^ **± se**		**Heterozygous sires (/6)**		**Fayoumi origins**^**b**^	
		**Significance level**	**QTL1**	**QTL2**	**QTL1**	**QTL2**	**QTL1**	**QTL2**	**QTL1**	**QTL2**
F1	21	*	19	25	0.66 ± 0.34	0.74 ± 0.22	6	5	3/6	3/5
LES	21	*	20	24	0.60 ± 0.31	0.52 ± 0.23	6	6	3/6	3/6

* = 5% chromosome-wide.

^a^substitution effect expressed in trait units (F1 = composite variable in arbitrary units; LES = cecal lesion score in arbitrary units).

^b^number of alleles associated with high performances in the Fayoumi line.

**Table 3:** Antagonist effects for QTL identified on GGA21

**Animal**	**F1 – QTL1**^**a**^	**F1 – QTL2**^**a**^	**LES – QTL1**^**a**^	**LES – QTL2**^**a**^
1	0.754	-0.918	-0.422	0.416
2	0.77	-0.846	-0.864	0.744
3	-0.268	0.352	0.406	-0.42
4	0.666	-0.862	-0.458	0.494
5	-0.304	0.11	0.328	-0.204
6	1.164	-0.746	-1.092	0.816

^a^substitution effect expressed in trait units (F1 = composite variable in arbitrary units; LES = cecal lesion score in arbitrary units).

### Factor analysis

One objective of this study was to find an index capable of explaining a large amount of the variance associated with resistance to coccidiosis. The new composite variable F1 was highly and positively correlated with WG, HEMA and PC and negatively correlated with LES (Table 4). About 74% of the original variance was explained by the composite variable F1 only. Considering single-QTL and multi-QTL analyses together, eight QTL for composite variable F1 were detected among which six co-localized with one or more QTL for the traits studied and the best example is the co-location of four QTL for the composite variable F1, HEMA, LES and PC on GGA19. Thus, using a composite variable like F1 is useful since it confirmed the detection of QTL regions that affected more than one trait i.e. on GGA19 and 24 and also captured part of the variability that was missed using single traits (GGA3, 9 and 21). However, while the composite variable F1 was associated with some of the QTL regions, it did not identify most of the regions identified in the single-QTL analysis. Thus, the composite variable F1 is not a sufficiently good index to explain the variance associated with resistance to coccidiosis.

**Table 4:** Correlations between the composite variable F1 and the traits investigated

**Trait**^**a**^	**F1**
WG	0.821
T°	0.119
LES	-0.555
HEMA	0.675
PC	0.846

^a^PC = plasma coloration at 4d post-inoculation (log_10_(optical density at 480 nm)); T° = rectal body temperature (°C) at 4d post-inoculation; HEMA = hematocrit level (%) at 4d post-inoculation; LES = lesion (scaled from 0 "no lesion" to 4 "most severe lesion") at 7d post-inoculation.

### Origin of QTL alleles

For all the QTL detected with QTLMap, the parental origin of the allele associated with the highest value of the trait (here, "strong QTL allele") was determined. Parental origins and additive effects that estimate the differences between the resistant Fayoumi and susceptible Leghorn lines are in Table 1. Animals severely affected by coccidiosis are expected to present high LES values but reduced values for HEMA, PC, WG and T°. Therefore, since the Fayoumi line is supposed to be more resistant to *E. tenella* than the Leghorn line, it is hypothesized that the Fayoumi alleles will be associated with high HEMA, PC, WG and T° values while the Leghorn alleles will be associated with high LES values. The composite variable F1 cannot be considered in this analysis, since it is impossible to predict which breed would have the favorable combination. In the single-QTL analyses, only one QTL was found to have all the high value alleles originating from the same breed. Indeed, all the favorable alleles of the QTL for WG on GGA1 originated from the Fayoumi line, as expected. For the other QTL detected in the single-QTL analyses, which all affected T°, most of the alleles that were associated with a high rectal body temperature originated from the Leghorn line, as expected given this breed’s susceptibility to coccidiosis. Multi-QTL analyses showed that the allele origins were equally shared between the Fayoumi and Leghorn lines and provided no further insight. Taken together, these results show that the QTL are not fixed in the founder lines (there is no QTL for which all F_1_ animals are heterozygous and all favorable alleles segregate from the same line) and confirm that most of the favorable alleles (i.e. associated to resistance performances) are segregating in the Fayoumi breed.

### Comparison of the models and analyses

Most of the QTL reported in this study are not described in the literature [20]. Twenty-eight chromosome-wide significant QTL (P < 0.05) were detected by applying interval mapping strategies with QTLMap and GridQTL. However, the use of a dense genetic map made it possible to test more complex hypotheses (multi-QTL analyses in QTLMap) and to map four new QTL.

This study was a follow-up to an experiment reported in [4], which applied the QTLexpress "F_2_" model and a selective genotyping strategy that used a low-density marker map comprising 157 microsatellites over 22 chromosomes. To compare the two studies, the locations of the markers from the first study were transposed to the genetic map used here.

Only three QTL i.e. for WG on GGA1, T° on GGA2 and HEMA on GGA6 were common between the two studies. The QTL carried by GGA3 at 227 cM can also be considered as common since it had an effect on the composite variable F1 in the present study and on WG and PC in the microsatellite-based study. If we consider only the results obtained by the GridQTL F_2_ model, only two common QTL are detected i.e. the QTL for T° on GGA2 and for HEMA on GGA6. Surprisingly, although our study included more markers and animals, the QTL significance was lower, which could be explained by the fact that using only extreme animals artificially increased the QTL effect and therefore the test value [21]. Taken together these results illustrate the differences in accuracy and robustness obtained when using the same model on 260 F_2_ animals genotyped for 157 microsatellites *versus* 860 F_2_ animals genotyped for 1393 genetic markers (including the157 microsatellites). Comparison of the results obtained with the QTLMap half-sib model and the GridQTL "F_2_" model on the full dataset (860 F_2_ individuals and 1393 genetic markers) revealed only one common QTL for T° on GGA2, although the QTL locations were slightly different. QTL were also identified at the same location on GGA22, but for different traits (LES with the QTLMap half-sib model and WG and composite variable F1 with the GridQTL "F_2_" model). This might correspond to a complex region regulating different traits, but only some of the traits are captured depending on the model used. In addition, both methods identified a QTL for WG on GGA1 but at different locations. However, the hypothesis that two different QTL for WG might be segregating on GGA1 was not confirmed under the multi-QTL analysis using QTLMap. The observed differences between these two models are clearly due to the different assumptions they are based on. The "F_2_" option of GridQTL assumes that QTL alleles are fixed in founder populations, whereas the QTLMap option "mixture of full and half-sib family" assumes that there are as many QTL alleles as founders. In addition, the probabilities of transmission are computed differently [22], and assumptions on both the QTL effect and residual variances (i.e. homoscedastic or heteroscedastic models) are also different. Differences in how the residual variance is modeled are shown by the average order of magnitude of additive values for the QTL detected, which is clearly influenced by the number of parameters to be estimated. It is worth noting that whatever the model or design (full or extremes) used, the QTL for T° on GGA2 is always detected and thus, can be considered a promising QTL for further investigation.

### Candidate genes

In order to identify possible candidate genes in the QTL regions detected, we used Ingenuity Pathway Analysis to derive functional annotations of these genes, with special emphasis on pathways or biological functions related to both the challenge applied for coccidiosis resistance and the traits measured, such as immune response, inflammatory response, response to infectious diseases, hematological system and carotenoid metabolism. The list of putative candidate genes identified (symbols and full names) [see Additional file 2: Table S2].

**Figure 1:**
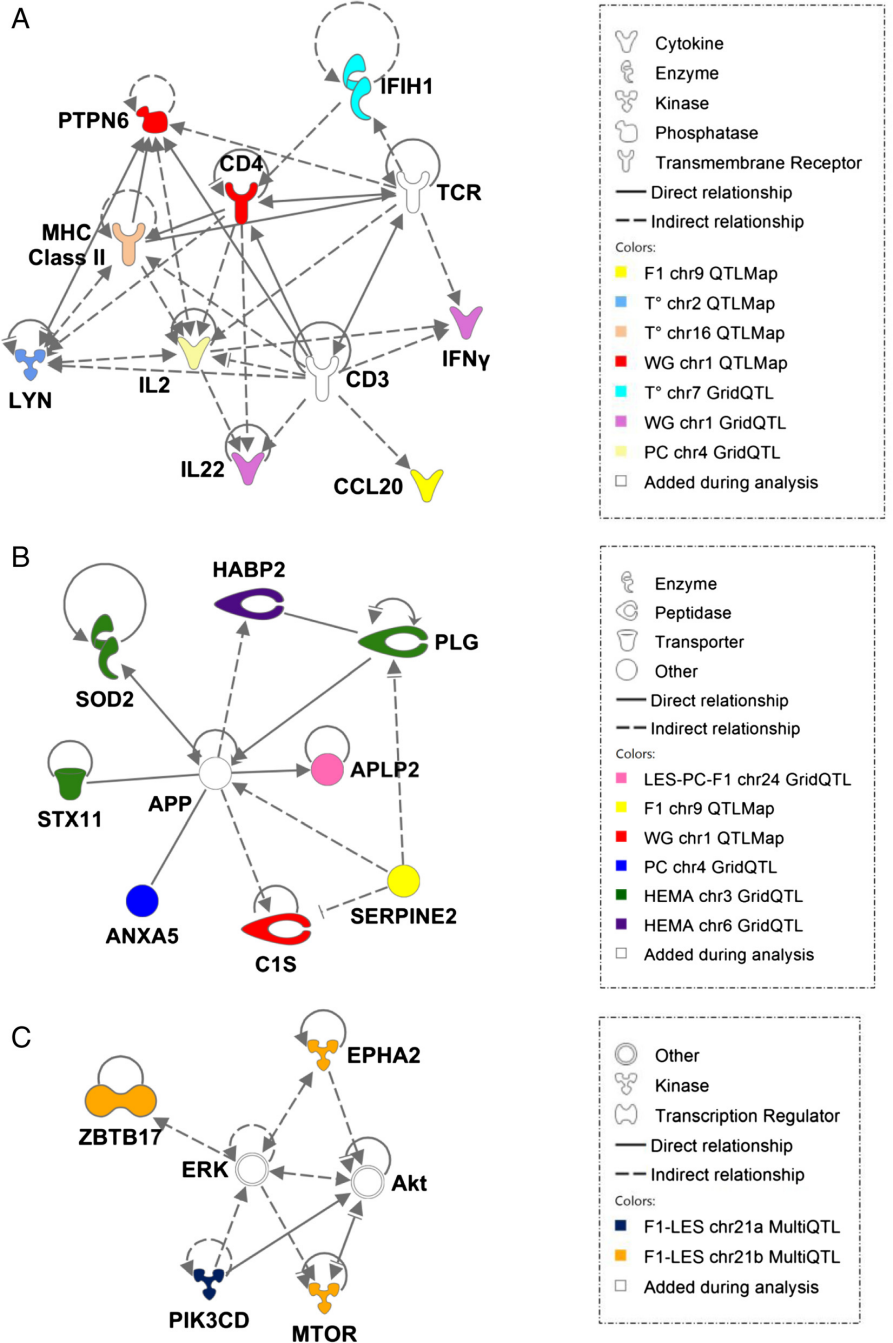
**Network of interactions between products of candidate genes from the QTL regions.** The candidate genes were identified as **(A)** involved in the pathway of immune response to infectious diseases, **(B)** involved in the coagulation pathway, and **(C)** candidate genes from the two QTL regions on chromosome GGA21 that was detected by multi-QTL screening; the network illustrates molecular interactions between the products of the candidate genes selected from the QTL regions; relations were determined using information contained in the IPA relationships database; the color code indicates the genes that are contained in a given QTL region; the white color indicates gene products that were added in the IPA analysis because of their interaction with the target gene products.

A network of interacting molecules involved in immune response to infectious diseases was generated and included eight genes (*IFIH1*, *IFNγ*, *CCL20*, *IL22*, *IL2*, *LYN*, *PTPN6*, *CD4*) and several genes of the *MHC* locus (Figure 1A) present in seven different QTL regions that were mainly related to weight gain and temperature. This network is centered both on the class II MHC pathway of antigen presentation, where the MHC class II molecules present extracellular peptides (such as the peptides derived from *Eimeria*) to the T-cell receptor (TCR) of CD4 T-cells, and on the pathway of the induction of cytokines like IFNγ. In the *MHC* locus, the genes directly involved in class II antigen presentation are *BLB1*, *BLB2* and *B-DM*[23]. This network of candidate genes is compatible with QTL regions involved in the pathway of presentation of *Eimeria*-derived antigens to initiate adaptive immune and inflammatory responses. Efficient induction of the immune response against *Eimeria tenella* by antigen-presenting cells has been demonstrated [24], as well as the role of IFNγ in increasing the weight of chicken infected by *Eimeria*[25].

Coccidiosis infection causes intestinal bleeding, particularly in the cecum and results in a decreased hematocrit count. We have identified a network of genes (*SOD2*, *STX11*, *PLG*, *ANXA5*, *C1S*, *APLP2*, *HABP2* and *SERPINE2*) present in six different QTL regions, centered on the amyloid beta (A4) precursor protein (APP; Figure 1B) and involved in blood clot fibrinolysis. In particular, SERPINE2 and APLP2 control the cleavage of plasminogen (PLG) that releases active fibrinolotytic plasmin. Moreover, the *HABP2* and *PLG* genes are present in QTL regions that affect HEMA.

Using a specific approach, we targeted the genes from the QTL regions identified for the composite variable F1 and the LES trait on chromosome GGA21 by the multi-QTL approach. As stated earlier, the hypothesis is that the region contains two closely-located QTL but in repulsion phase, which is compatible with a scenario in which two candidate genes interact in the same functional network. Among the genes present in these two QTL regions, we identified four putative candidate genes, three being located in the same QTL region (Figure 1C). *MTOR* and *PIK3CD*, which code for proteins that interact directly with AKT and indirectly with ERK, two protein families involved in signaling pathways, among which the PIK3-Akt-mTOR pathway is crucial for lymphocyte development. Interestingly, it has recently been shown in humans that gain-of-function mutations in the gene *PIK3CD* induced phosphorylation of AKT and activation of mTOR, leading to premature T-cell senescence and immunodeficiency [26].

Many of the genes involved in carotenoid biosynthesis/catabolism were contained in some of the QTL regions detected: four genes from the retinol-binding protein (RBP) family (*RBP1*, *RBP2*, *RBP5*, *RBP7*), *BCMO1*, *RDH10*, *ALDH1A3* and *SDR16C5*. All these genes may have a role on PC, which reflects the amount of carotenoids in the plasma. Note that *BCMO1* is also known to be involved in the determinism of chicken muscle yellow color [27].

This approach based on the functional investigation of genes from different QTL regions narrows down the list of putative candidate genes by exploiting their documented interactions in biological pathways related to the study.

## Competing interests

The authors declare that they have no competing interests.

## Authors’ contributions

NB and OD carried out the QTL mapping analyses. BB validated the genotyping data and contributed to data interpretation. OF and HR contributed to the statistical analyses. DG managed the animal production and phenotyping phases. JMR was in charge of the coccidiosis challenge. PLR contributed to data interpretation. MHP and OD managed the funding and design of the experiment. OD, BB and MHP drafted the manuscript. All authors have read and approved the final manuscript.

## Authors’ information

Nicola Bacciu and Bertrand Bed’Hom are co-first authors.

## Supplementary Material

Additional file 1: Table S1 Genetic map. Location on the genome of all the genetic markers genotyped on the F_2_ design. Click here for file

Additional file 2: Table S2 List of genes in QTL regions. All genes located in QTL regions were extracted using AnnotQTL. Putative candidate genes based on functional analysis (using IPA) are flagged in green. Putative candidate genes involved in networks of interactions between products from genes located in different QTL regions are flagged in red. Click here for file
